# The effect of an e-learning course on nursing staff’s knowledge of delirium: a before-and-after study

**DOI:** 10.1186/s12909-015-0289-2

**Published:** 2015-02-05

**Authors:** Lotte van de Steeg, Roelie IJkema, Cordula Wagner, Maaike Langelaan

**Affiliations:** 1 NIVEL Netherlands institute for health services research, Utrecht, the Netherlands; 2 Present affiliation: Erasmus Medical Centre, Rotterdam, the Netherlands; 3 EMGO Institute for Health and Care Research, VU University Medical Centre, Amsterdam, the Netherlands

**Keywords:** Delirium, Education, Nurses, e-learning

## Abstract

**Background:**

Delirium is a common condition in hospitalized patients, associated with adverse outcomes such as longer hospital stay, functional decline and higher mortality, as well as higher rates of nursing home placement. Nurses often fail to recognize delirium in hospitalized patients, which might be due to a lack of knowledge of delirium diagnosis and treatment. The objective of the study was to test the effectiveness of an e-learning course on nurses’ delirium knowledge, describe nursing staff’s baseline knowledge about delirium, and describe demographic factors associated with baseline delirium knowledge and the effectiveness of the e-learning course.

**Methods:**

A before-and-after study design, using an e-learning course on delirium. The course was introduced to all nursing staff of internal medicine and surgical wards of 17 Dutch hospitals.

**Results:**

1,196 invitations for the e-learning course were sent to nursing staff, which included nurses, nursing students and healthcare assistants. Test scores on the final knowledge test (mean 87.4, 95% CI 86.7 to 88.2) were significantly higher than those on baseline (mean 79.3, 95% CI 78.5 to 80.1). At baseline, nursing staff had the most difficulty with questions related to the definition of delirium: what are its symptoms, course, consequences and which patients are at risk. The mean score for this category was 74.3 (95% CI 73.1 to 75.5).

**Conclusions:**

The e-learning course significantly improved nursing staff's knowledge of delirium in all subgroups of participants and for all question categories. Contrary to other studies, the baseline knowledge assessment showed that, overall, nursing staff was relatively knowledgeable regarding delirium.

**Trial registration:**

The Netherlands National Trial Register (NTR). Trial number: NTR 2885, 19 April 2011.

## Background

Delirium is a common condition in hospitalized people, with the highest incidence rates reported in intensive-care, palliative care and postoperative care settings [1-4]. It is a neuropsychiatric syndrome characterized by a disturbance of consciousness and attention, as well as a change in cognition or a disturbance of perception [5,6]. Delirium is associated with adverse outcomes, such as longer hospital stay, functional decline and increased mortality, as well as higher rates of nursing home placement [3,7-9]. Previous research has shown that nurses often fail to recognize delirium in hospitalized patients [10,11] limiting their ability to provide adequate delirium care. Several authors have suggested that this poor recognition is caused by a lack of knowledge of delirium, especially of the importance of early recognition and the treatment of delirium [10,12-14]. Increasing nursing knowledge of delirium through educational programmes could improve nurses’ identification of delirious patients and the quality of delirium care they provide.

Previous studies have examined nurses’ level of knowledge regarding delirium. Hare et al. [15], for example, assessed nurses’ level of knowledge using a questionnaire including questions on delirium and its associated risk factors. They concluded that nurses had inadequate levels of knowledge, particularly in relation to risk factors. Other recent studies examined the effect of an educational intervention on nurses’ knowledge of delirium [16-19]. These showed that an educational intervention on delirium significantly improved nurses’ knowledge. Furthermore, Meako et al. [17] found that the number of years of experience of nurses significantly influenced the effect of an educational intervention on test scores, while the level of education did not. However, these studies were limited by the use of a small number of hospitals (≤3), a small sample size, and in all but one study a low number of items in the delirium knowledge tests. Therefore, further insight into differences in knowledge level and the extent to which subgroups of nurses benefit from educational interventions is needed. This will provide hospitals with the information needed to tailor their educational policy for specific subgroups.

In the context of quality and safety in hospitals, it is important to guarantee a sufficient level of knowledge in health professionals. As an alternative to traditional education, which is relatively labour-intensive and time-consuming, e-learning can be useful when improving knowledge in a large group of nurses [20]. Advantages of e-learning include flexibility in time management, pace of learning, and quality assurance. However, barriers such as a limited level of computer literacy in users are also associated with the use of e-learning [21,22].

In this study we investigated the effect of an e-learning course, based on nationally recommended guidelines for delirium [23,24], on nurses’ delirium knowledge. Furthermore, we examined the baseline delirium knowledge in a large sample of nurses working in hospitals throughout the Netherlands. In addition, we described the association between participant demographics and baseline delirium knowledge, as well as between demographics and the effect of the course on this knowledge.

## Methods

### Study design and population

An e-learning course on delirium was introduced to the hospitals participating in a trial measuring the effect of e-learning on the implementation of a quality improvement project with special attention for delirium care, which has been described extensively elsewhere [25,26]. One hospital included initially refrained from participation due to practical circumstances in the hospital. The remaining 17 hospitals included two university hospitals, four tertiary teaching hospitals and 11 general hospitals. Each hospital participated in the study with two wards; typically these would be an internal medicine ward and a surgical ward. All nursing staff working on these wards were invited to participate in the study, including healthcare assistants and nursing students participating in a clinical placement at the hospital.

The moment each hospital received access to the intervention was randomized: the first hospital started in June 2011, the last hospitals started in March 2012. Each month, one or two hospitals gained access to the intervention, resulting ultimately in all hospitals having had access to the intervention. Contact persons from the hospitals provided the researchers with names, email addresses and demographic characteristics of all nursing staff working on the participating wards. Delirium knowledge was tested before and after participants completed the e-learning course.

### Intervention

The e-learning course was developed by a commercial publisher [27] in collaboration with a Dutch hospital. The course was reviewed, while still in development, by the researchers and by the Netherlands Centre of Excellence in Nursing. The content of the e-learning course was consistent with Dutch guidelines regarding delirium care [23,24]. It consisted of a baseline knowledge test, the course itself, and a final knowledge test.

The aims of the e-learning course were to create or increase awareness about delirium and the associated risks, and to increase knowledge about delirium care. It incorporated case studies and short tests for self-assessment to facilitate the learning experience. The course contained information on subjects such as clinical features, risk factors, diagnostics, prevention and treatment (Table 1). The estimated time needed to complete the course, including both knowledge tests, was four hours.

**Table 1:** **Content of the delirium e-learning course**

Chapter	Content
I. Introduction	i. Introduction to the e-learning course, the patients from the case studies and the subject
II. What is delirium?	i. Introduction to the goals and content of the chapter
	ii. Definition of delirium, its clinical features and course
	iii. Risk patients, predisposing and precipitating risk factors, and prevention
	iv. Consequences of delirium
III. Risk screening	i. Introduction to the goals and content of the chapter
	ii. Predisposing and precipitating risk factors and risk screening
	iii. Recording and discussing delirium risk of a patient
IV. Preventive interventions	i. Introduction to the goals and content of the chapter
	ii. Short overview of preventive medical interventions
	iii. Preventive nursing interventions
V. Early recognition and diagnostics	i. Introduction to the goals and content of the chapter
	ii. The importance of early recognition of delirious patients
	iii. Delirium Observation Screening scale
	iv. Confusion Assessment Method - ICU
	v. Delirium and dementia, delirium tremens and delirium caused by medication
VI. Treatment and care	i. Introduction to the goals and content of the chapter
	ii. Focus of treatment and disciplines involved
	iii. Medical treatment
	iv. Nursing interventions regarding treatment and care
	v. Aftercare
	vi. Delirium in the terminal or palliative phase
VII. More information	i. References to guidelines, reports and other sources of information on delirium

In turn, each hospital gained a 3-month period access to the e-learning course. Access codes and instructions were sent to the nursing staff of participating wards by email, on the first day of access. In addition, a meeting was organized in each hospital to introduce the course, to explain the goals of the research project, and to answer any questions. After each month, participants who had not yet completed the course received a reminder by email from the researchers. Furthermore, contact persons from the hospitals received a monthly overview of the degree of participation in their wards. Nursing staff were able to access the e-learning course from any computer with internet access, which gave them the opportunity to choose whether to follow the course at home or at work.

### Knowledge tests

Prior to starting the course, nurses had to take a baseline knowledge test consisting of a random sample of 24 questions - multiple choice, true/false and matching questions - out of a database with 82 different questions about delirium and delirium care. The questions in the database used in the study were developed by Leerstation Zorg [28], which is a national foundation that develops tests and test questions for organizations in healthcare. This development is always carried out by a group of experts, including a specialist in educational measurement, two to ten experts on the subject matter - such as physicians and nurses - and a text editor. While the specialist in educational measurement is responsible for ensuring construct validity of the tests and the questions, the experts on the subject matter are responsible for ensuring content validity. To further guarantee sufficient validity and reliability, all test questions are put through a validation process before being used regularly. This validation process is carried out by a panel of 20 to 50 people selected from the intended user group, who answer the questions and comment on the content or phrasing of the questions. These answers and comments are analysed and used by the expert group to adjust questions if necessary. The use of experts and a test panel ensure that the questions correspond with the level of knowledge in the intended user group. The cut-off score of each test - the lowest possible score that must be earned to pass the test – is calculated, taking into account the possibility that a question can be answered correctly by chance. Finally, the validity of each question is tested after the first 50 times a question is used and again after the first 200 times.

The questions in the delirium knowledge test were divided into five categories, with a predetermined number of questions from each category being combined to form one test: definition (five questions), risk screening and prevention (seven questions), early recognition (four questions), Delirium Observation Screening scale [29] (DOS scale) (two questions) and treatment (six questions). After completing the e-learning course, participants were asked to take a final knowledge test, which again consisted of 24 randomly selected questions. The participants could choose when to take the final test: it was possible to take the test immediately after completing the e-learning course, or to take the test at a later date. However, unlike the e-learning course, the test had to be completed in one sitting. When successfully completing this final test by answering 80% or more of the questions correctly, participants were provided with a certificate which could be used for applying for quality registry accreditation points. The e-learning and final test were only accessible after starting the baseline knowledge test.

### Statistical analysis

All statistical analyses were performed using STATA 12.1 and MLwiN 2.25. Demographics of participants and non-participants were compared using the student’s *t*-test or Chi squared test. Multivariable multilevel linear regression analysis - including hospital level, ward level, nurse level, test level - was used to compare mean baseline test scores and change scores within subgroups. These analyses were adjusted for repeated measures. Analyses of the baseline and final test score per question category were also conducted using multivariable multilevel linear regression analysis, adjusted for repeated measures. When analyzing the change scores, all completed tests were included: when a nurse did not have a complete baseline test and a complete final test, the one test that was available was included in the analysis. In order to gain insight into the effect size, Cohen’s *d* was calculated, by dividing the change score (the mean of the final score minus the mean of the baseline score) by the standard deviation of the baseline score.

### Ethics

The study had been granted ethical approval by the ethical review board of VU University Medical Center in Amsterdam, the Netherlands, which covered the study in all participating hospitals.

## Results

All nursing staff working on the participating hospital wards received an invitation to participate in the e-learning course, resulting in 1,196 invitations being sent. The mean age of invited nursing staff was 35.7 years, 7.4% were male. The largest group comprised nurses (95.7%), which included ward managers, regular nurses, and specialized nurses. Almost a quarter of the included nursing staff had a bachelor’s or master’s degree in nursing and 62.4% worked in a general hospital.

**Figure 1:**
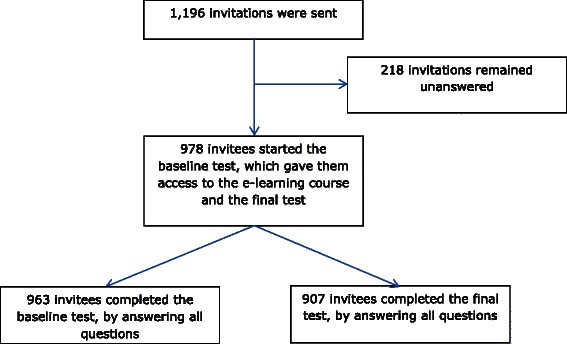
**Flow chart to illustrate the response rate of nursing staff.**

978 invitees started the baseline test, resulting in a participation rate of 86.4% (95% CI 81.1 to 90.5), adjusted for clustering on the ward and hospital level. Characteristics of participants as well as non-participants are shown in Table 2. Participants were on average slightly older than non-participants (36.1 vs. 33.8 years). The number of people younger than 30 years was higher in the non-participant group (48.4%) compared to the participant group (35.5%) (p < 0.01). Furthermore, participants were less often nursing students (0.9 % vs. 5.0%) (p < 0.01), and worked more often on an internal medicine ward (51.0% vs. 41.7, <0.01). Incomplete tests - where not all 24 test questions were answered - were excluded from further analysis (see Figure 1).

**Table 2:** **Characteristics of the research population**

	Participants (%) (n = 978)	Non-participants (%) (n = 218)	P value
**Age** (202 missing values)			
<30 years	297 (35.5)	76 (48.4)	<0.01
30-50 years	395 (47.2)	58 (36.9)	
>50 years	145 (17.3)	23 (14.7)	
**Sex** (69 missing values)			
Male	63 (6.8)	20 (10.2)	0.09
Female	868 (93.2)	176 (89.8)	
**Function** (1 missing value)			
Nurse	945 (96.7)	199 (91.3)	<0.01
Nursing student	9 (0.9)	11 (5.0)	
Healthcare assistant	23 (2.3)	8 (3.7)	
**Level of education** (216 missing values)			
Vocational	634 (75.8)	107 (74.3)	0.69
Bachelor or master	202 (24.2)	37 (25.7)	
**Experience** (233 missing values)			
0-1 years	33 (4.1)	13 (8.4)	0.09
1-5 years	362 (44.8)	69 (44.8)	
5-10 years	171 (21.1)	34 (22.1)	
>10 years	243 (30.0)	38 (24.7)	
**Type of ward** (0 missing values)			
Internal medicine ward	499 (51.0)	91 (41.7)	<0.01
Surgical ward	414 (42.3)	119 (54.6)	
Other	65 (6.7)	8 (3.7)	
**Type of hospital** (0 missing values)			
University	114 (11.7)	18 (8.3)	0.10
Tertiary teaching	249 (25.5)	69 (31.7)	
General	615 (63.9)	131 (60.1)

Participants’ mean score for the baseline test was 79.3 (95% CI 78.5 to 80.1, SD 10.5, n = 650) on a scale of 0 to 100, adjusted for age, sex, function, level of education, experience, type of ward and type of hospital. Those aged 50 years or older scored significantly lower than those under the age of 30 (77.0 vs. 79.7, p < 0.01) (Table 3) and those aged between 30 and 50 (79.7, p < 0.01). Furthermore, nursing staff with a bachelor’s or master’s degree scored significantly higher than those with a vocational education (81.2 vs. 78.7, p < 0.01). No significant differences were found for sex, function, work experience, type of ward or type of hospital. After following the e-learning course, 907 of the 978 participants - 95.4% (95% CI 92.3 to 97.3) - continued to complete the final test. Their mean final test score was 87.4 (95% CI 86.7 to 88.2, n = 618), adjusted for age, sex, function, level of education, experience, type of ward and type of hospital. On average, final test scores were 8.1 points higher than baseline test scores (95% CI 7.4 to 8.8, p <0.01), resulting in an effect size of 0.8. The change scores by demographic category are shown in Table 4. No significant differences in change score were found between subgroups.

**Table 3:** Baseline test score by demographic category, using multivariable multilevel linear regression analysis*

	Mean score	95% CI	P value
**Age (year)**			
<30	79.7	78.5 to 80.9	r.c.
30-50	79.8	78.8 to 80.8	0.84
>50	**77.0**	**75.4 to 78.7**	**<0.01**
**Sex**			
Male	79.4	77.1 to 81.7	r.c.
Female	79.3	78.5 to 80.1	0.92
**Function**			
Nurse	79.4	78.5 to 80.2	r.c.
Nursing student	78.3	72.1 to 84.5	0.74
Healthcare assistant	77.5	73.7 to 81.3	0.34
**Level of education**			
Vocational	78.7	77.9 to 79.6	r.c.
Bachelor or master	**81.2**	**79.9 to 82.6**	**<0.01**
**Experience (year)**			
0-1	77.4	74.5 to 80.2	r.c.
1-5	79.5	78.4 to 80.6	0.15
5-10	78.6	77.2 to 80.0	0.45
>10	79.9	78.7 to 81.2	0.10
**Type of ward**			
Internal medicine ward	79.4	78.4 to 80.4	r.c.
Surgical ward	79.3	78.2 to 80.3	0.86
Other	73.4	64.7 to 82.1	0.18
**Type of hospital**			
University	79.5	77.3 to 81.6	r.c.
Tertiary teaching	79.7	77.9 to 81.6	0.86
General	79.1	78.1 to 80.1	0.79

*Incomplete tests were excluded from analysis. Bold: test score differs significantly from the test score of the reference category.

**Table 4:** Change score by demographic category, using multivariable multilevel linear regression analysis*

	Mean	95% CI	P value
**Age (year)**			
<30	8.2	7.1 to 9.4	r.c.
30-50	7.7	6.7 to 8.7	0.49
>50	9.3	7.5 to 11.0	0.33
**Sex**			
Male	6.9	4.3 to 9.5	r.c.
Female	8.2	7.5 to 8.9	0.33
**Function**			
Nurse	8.0	7.3 to 8.8	r.c.
Nursing student	12.7	5.5 to 19.8	0.20
Healthcare assistant	9.6	5.0 to 14.2	0.51
**Level of education**			
Vocational	8.4	7.6 to 9.2	r.c.
Bachelor or master	7.1	5.6 to 8.6	0.12
**Experience (year)**			
0-1	9.7	6.5 to 12.9	r.c.
1-5	7.7	6.6 to 8.8	0.25
5-10	8.5	7.0 to 10.0	0.51
>10	8.2	7.0 to 9.5	0.42
**Type of ward**			
Internal medicine ward	8.0	7.1 to 9.0	r.c.
Surgical ward	8.1	7.1 to 9.2	0.92
Other	17.7	7.6 to 27.7	0.06
**Type of hospital**			
University	9.2	7.3 to 11.2	r.c.
Tertiary teaching	7.2	5.7 to 8.6	0.09
General	8.3	7.4 to 9.1	0.36

*Incomplete tests were excluded from analysis.

When comparing different categories of questions, we found that participants scored best at baseline on questions from the category Delirium Observation Screening scale (DOS scale) (83.8, p < 0.05) (Table 5). Lowest scores at baseline were found for the question category Definition (74.3, p < 0.05). However, change scores were highest for this category (11.3, p < 0.01). For all question categories, we found a significantly higher score on the final test compared to the baseline test.

**Table 5:** Test scores and change score per question category, using multivariable multilevel linear regression analysis*

Category	Score baseline test (95% CI)	Score final test (95% CI)	Change score (95% CI)
**Definition**	74.3 (73.1 to 75.5)	85.6 (84.5 to 86.6)	11.3 (9.7 to 12.8)
**Risk screening and prevention**	81.4 (80.3 to 82.5)	89.5 (88.6 to 90.5)	8.1 (7.1 to 9.2)
**Early recognition**	78.0 (76.7 to 79.3)	83.2 (81.9 to 84.5)	5.2 (3.4 to 7.0)
**DOS scale**	83.8 (82.1 to 85.6)	89.6 (88.2 to 91.1)	5.8 (4.2 to 7.5)
**Treatment**	76.5 (75.1 to 77.9)	84.1 (82.8 to 85.3)	7.5 (5.9 to 9.2)

*Incomplete tests were excluded from analysis.

## Discussion

In this study we investigated the effect of e-learning on specific aspects of delirium knowledge in nursing staff in hospitals in the Netherlands. We found that the e-learning course had a significant positive effect on nurses’ knowledge on delirium, in all subgroups of nursing staff and for all question categories, with a mean change score of 8.1 and a large effect size (0.8). At baseline, nursing staff had the most difficulty with questions related to the definition of delirium: what are its symptoms, course, consequences, and which patients are at risk.

Of the 1,196 members of nursing staff that were invited to participate, 978 started the baseline knowledge test. This high level of participation might confirm the findings of Karaman [30], who found that nurses were open to online learning and found it to be appropriate for their working conditions and needs. However, we expect that in this study the email reminders and encouragement provided by team leaders following the monthly overview of participation per ward increased participation rates. Nursing staff’s baseline knowledge was fairly high, with a mean test score of 79.2, where other studies found a mean score of 53-61 [15-17]. This could indicate that more attention has been paid to delirium in nursing education in the Netherlands than in other countries. However, it might not be possible to directly compare the test scores from these different studies, because they all used different instruments to test the level of delirium knowledge. Future research should focus on utilizing the same assessment instrument on delirium knowledge in different studies and in different countries. This would not only make it possible to compare studies and educational interventions, but would also provide insight into the differences in levels of delirium knowledge between countries with different health care systems and educational systems.

Some significant differences were found in the current study between baseline test scores of different demographic groups. Nursing staff aged over 50 had a lower average baseline score than their younger colleagues. Furthermore, nursing staff with a bachelor’s or master’s degree had a significantly higher baseline score than staff with a vocational education. However, these differences in scores were relatively small. The differences between older and younger members of nursing staff might indicate that the education on delirium that nursing students receive has improved in the Netherlands over the years. Because the course was originally developed for nursing staff with a bachelor’s degree, we might have expected nurses with a bachelor’s or master’s to benefit more from the e-learning course than those with a vocational education. This, however, was not the case. Our study shows that, regardless of characteristics of the individual nurse, on average nurses benefitted significantly from the e-learning course. While Meako et al. [17] also found that an educational intervention on delirium was effective for all participating nurses, no matter their educational level or years of experience, they did find that those nurses with little work experience benefitted more from the e-learning. Our findings did not show a similar impact of work experience on the effect of e-learning on knowledge.

At baseline, nursing staff had the most difficulty with questions related to the definition of delirium. However, this was also the question category that saw the highest knowledge increase. This indicates that e-learning was effective in decreasing a knowledge gap that existed among the participating nursing staff. Besides the questions on definition, nursing staff also scored relatively low on the questions regarding treatment of delirium. These findings are similar to those of Agar et al., in Australia, who found that nurses had limited knowledge of the features of delirium, as well as delirium management [31].

The strengths of this study are its large sample size, the relatively large number of questions per test and the use of an e-learning course developed by a group of experts on didactics as well as content. However, our study also has some limitations. First, because the final knowledge test was taken online, the possibility exists that participants used written notes or other sources of information in order to answer the test questions. There were no means available to the researchers to ensure nursing staff only used their own knowledge when taking the test. However, the method used in this study - with an online knowledge test and a certificate when successfully completing the test - is similar to how e-learning is used by Dutch hospitals in daily practice. Second, the current study only examined delirium knowledge of nursing staff shortly after completing the e-learning course. This means that no insight into knowledge retention was gained in this study, which would be important information for hospitals planning their education of nursing staff. Third, because the trial of which this study was a part followed a stepped wedge design, the e-learning course was eventually introduced in all participating hospital wards [25]. This means the effect on knowledge was tested using a before-and-after study design, without a control group. Fourth, while the e-learning course used in this study was based on the Dutch guidelines on delirium care available at the time, in 2013 a revision of the delirium guideline from 2004 was published [32]. This could indicate that the e-learning course is no longer up-to-date for the Dutch situation. However, the main changes made to the guideline regarding nursing care in hospitals concern an increased emphasis on non-pharmacological interventions, screening of patients for delirium risk factors and using the Delirium Observation Screening scale. These are all topics that were already included in the e-learning course, suggesting the course is still suitable for use.

## Conclusions

E-learning appears to have a positive effect on nursing staff’s knowledge of delirium, for all subgroups studied. Moreover, the high participation rate in our study appears to show that staff members are willing to improve their knowledge through e-learning. This, together with a high prevalence of delirium in hospitalized older patients, associated with adverse outcomes, advocates for a more widespread introduction of e-learning on delirium to nursing staff working in hospitals. When nursing staff have a better understanding of delirium and delirium care, they will be better able to recognize the importance of early detection of delirium and take measures to identify delirious patients or patients at risk for delirium. Increased knowledge on delirium care will also enable nursing staff to potentially prevent the occurrence of delirium and its negative consequences.

## Abbreviation

DOS scale: Delirium observation screening scale

## References

[1] Inouye S, Westendorp RGJ, Saczynski JS (2014). Delirium in elderly people. Lancet.

[2] Morandi A, Jackson JC (2011). Delirium in the intensive care unit: a review. Neurol Clin.

[3] Siddiqi N, House AO, Holmes JD (2006). Occurrence and outcome of delirium in medical in-patients: a systematic literature review. Age Ageing.

[4] Young J, Inouye SK (2007). Delirium in older people. Br Med J.

[5] Korevaar JC, van Munster BC, de Rooij SE (2005). Risk factors for delirium in acutely admitted elderly patients: a prospective cohort study. BMC Geriatr.

[6] Mittal V, Muralee S, Williamson D, McEnerney N, Thomas J, Cash M (2011). Review: Delirium in the elderly: a comprehensive review. Am J Alzheimers Dis Other Demen.

[7] Eeles EMP, Hubbard RE, White SV, O’Mahony MS, Savva GM, Bayer AJ (2010). Hospital use, instituationalisation and mortality associated with delirium. Age Ageing.

[8] Dasgupta M, Brymer C (2014). Prognosis of delirium in hospitalized elderly: worse than we thought. Int J Geriatr Psychiatry.

[9] Witlox J, Eurelings LSM, de Jonghe JFM, Kalisvaart KJ, Eikelenboom P, van Fool WA (2010). Delirium in elderly patients and the risk of postdischarge mortality, institutionalization, and dementia. JAMA.

[10] Flagg B, Cox L, McDowell S, Mwose JM, Buelow JM (2010). Nursing identification of delirium. Clinical Nurse Specialist.

[11] Rice KL, Bennett M, Gomez M, Theall KP, Knight M, Foreman MD (2011). Nurses’ recognition of delirium in the hospitalized older adult. Clinical Nurse Specialist.

[12] M El Hussein, S Hirst, Salyers V. Factors that contribute to underrecognition of delirium by registered nurses in acute care settings: a scoping review of the literature to explain this phenomenon. J Clinical Nursing. 2014. doi:10.1111/jocn.12693. [Epub ahead of print] 10.1111/jocn.12693 25293502

[13] Hosie A, Lobb E, Agar M, Davidson PM, Philips J. Identifying the barriers and enablers to palliative care nurses’ recognition and assessment of delirium symptoms: A qualitative study. J Pain and Symptom Management. 2014 doi:10.1016/j.jpainsymman.2014.01.008 [Epub ahead of print]. 10.1016/j.jpainsymman.2014.01.008 24726761

[14] Teodorczuk A, Reynish E, Milisen K (2012). Improving recognition of delirium in clinical practice: a call for action. BMC Geriatr.

[15] Hare M, Wynaden D, McGowan S, Landsborough I, Speed G (2008). A questionnaire to determine nurses’ knowledge of delirium and its risk factors. Contemp Nurse.

[16] Gesin G, Russell BB, Lin AP, Norton HJ, Evans SL, Devlin JW (2012). Impact of a delirium screening tool and multifaceted education on nurses’ knowledge of delirium and ability to evaluate it correctly. Am J Critical Care.

[17] Meako ME, Thompson HJ, Cochrane BB (2011). Orthopaedic nurses’ knowledge of delirium in older hospitalized patients. Orthop Nurs.

[18] McCrow J, Sullivan KA, Beattie ER (2014). Delirium knowledge and recognition: a randomized controlled trial of a web-based educational intervention for acute care nurses. Nurse Educ Today.

[19] Wand APF, Thoo W, Sciuriaga H, Ting V, Baker J, Hunt GE (2014). A multifaceted educational intervention to prevent delirium in older inpatients: a before and after study. Int J Nurs Stud.

[20] Lahti M, Hatonen H, Valimaki M (2014). Impact of e-learning on nurses’ and student nurses knowledge, skills, and satisfaction: a systematic review and meta-analysis. Int J Nurs Stud.

[21] McVeigh H (2009). Factors influencing the utilisation of e-learning in post-registration nursing students. Nurse Educ Today.

[22] Roe D, Carley S, Sherratt C (2010). Potential and limitations of e-learning in emergency medicine. Emerg Med J.

[23] VMS (National Patient Safety Programme). Kwetsbare ouderen. http://www.vmszorg.nl/Themas/Kwetsbare-ouderen.

[24] Van der Mast RC, Huyse FJ, Droogleever Fortuijn HA, Heeren TJ, Izaks GJ, Kalisvaart CJ (2004). Guideline delirium.

[25] Van de Steeg L, Langelaan M, Ijkema R, Wagner C (2012). The effect of a complementary e-learning course on implementation of a quality improvement project regarding care for elderly patients: a stepped wedge trial. Implement Sci.

[26] Van de Steeg L, Ijkema R, Langelaan M, Wagner C (2014). Can an e-learning course improve nursing care for older people at risk of delirium: a stepped wedge cluster randomised trial. BMC Geriatr.

[27] Noordhoff Publishers. Cursusaanbod; Cursus Delier. [http://www.noordhoff-health.nl/wps/portal/zorg/e-learning-ziekenhuizen-verpleegkundige/cursusaanbod/cursussen/patientveiligheid/cursus-delier] In Dutch.

[28] Leerstation zorg. Over leerstation zorg; Het proces. [http://www.leerstationzorg.nl] In Dutch.

[29] Van Gemert LA, Schuurmans MJ (2007). The Neecham Confusion Scale and the Delirium Observation Screening Scale: capacity to discriminate and ease of use in clinical practice. BMC Nurs.

[30] Karaman S (2011). Nurses’ perceptions of online continuing education. BMC Med Educ.

[31] Agar M, Draper B, Philips PA, Philips J, Collier A, Harlum J, Currow D (2012). Making decisions about delirium: A qualitative comparison of decision making between nurses working in palliative care, aged care, aged care psychiatry, and oncology. Palliat Med.

[32] Dautzenberg PLJ, Molag ML, van Munster BC, de Rooij SEIA, Luijendijk HJD, Leentjes AFG (2014). Herziene richtlijn ‘Delier volwassenen en ouderen’. Ned Tijdschr Geneeskd.

